# Early Diagnosis of Colorectal Cancer Based on Bisulfite‐free Site‐specific Methylation Identification PCR Strategy: High‐Sensitivity, Accuracy, and Primary Medical Accessibility

**DOI:** 10.1002/advs.202401137

**Published:** 2024-06-13

**Authors:** Linqing Zhen, Xinlu Tang, Zhengguo Xu, Yizhou Huang, Xiaohua Qian, Haiping Lin, Chao Li, Rong Cui, Hongsheng Fang, Hao Yang, Jiani Qiu, Zhaoqi Fang, Xiaohuan Peng, Yifeng Jin, Jianing Nie, Shiwei Guo, Yuguang Wang, Ming Zhong, Hongchen Gu, Hong Xu

**Affiliations:** ^1^ School of Biomedical Engineering Shanghai Jiao Tong University Shanghai 200030 P. R. China; ^2^ Hefei Cancer Early Screening Innovation Technology Institute Anhui Province China; ^3^ Medical community of Linhai First People's Hospital Zhejiang 317000 P. R. China; ^4^ Renji Hospital Affiliated to Shanghai Jiao Tong University School of Medicine Shanghai 200030 P. R. China; ^5^ Jiading Hospital of Traditional Chinese medicine Shanghai 201800 P. R. China; ^6^ Department of Colorectal Surgery Fudan University Shanghai Cancer Center Shanghai 200032 P. R. China; ^7^ Shanghai Healzone Biotechnology Co., LTD Shanghai 200000 P. R. China

**Keywords:** ColoC‐mSTEM, Colorectal cancer, early detection, primary medical accessibility, specific methylation site

## Abstract

Due to its decade‐long progression, colorectal cancer (CRC) is most suitable for population screening to achieve a significant reduction in its incidence and mortality. DNA methylation has emerged as a potential marker for the early detection of CRC. However, the current mainstream methylation detection method represented by bisulfite conversion has issues such as tedious operation, DNA damage, and unsatisfactory sensitivity. Herein, a new high‐performance CRC screening tool based on the promising specific terminal‐mediated polymerase chain reaction (STEM‐PCR) strategy is developed. CRC‐related methylation‐specific candidate CpG sites are first prescreened through The Cancer Genome Atlas (TCGA) and Gene Expression Omnibus (GEO) databases using self‐developed bioinformatics. Next, 9 homebrew colorectal cancer DNA methylated STEM‒PCR assays (ColoC‐mSTEM) with high sensitivity (0.1%) and high specificity are established to identify candidate sites. The clinical diagnostic performance of these selected methylation sites is confirmed and validated by a case‐control study. The optimized diagnostic model has an overall sensitivity of 94.8% and a specificity of 95.0% for detecting early‐stage CRC. Taken together, ColoC‐mSTEM, based on a single methylation‐specific site, is a promising diagnostic approach for the early detection of CRC which is perfectly suitable for the screening needs of CRC in primary healthcare institutions.

## Graphical abstract

ColoC‐mSTEM is a site‐specific methylation detection strategy developed for the early detection of CRC based on bisulfite‐free STEM‐PCR technology. This method is characterized by high sensitivity, high accuracy, and easy operation, and the sensitivity for detecting CRC is 94.1% with a specificity of 95.0%. The method is perfectly suitable for the screening needs of CRC in primary healthcare institutions.

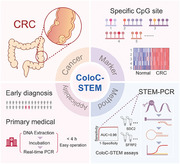

## 1 Introduction

Colorectal cancer (CRC) is one of the most common malignant tumors in the world; it has the third‐highest incidence rate and second‐highest mortality rate among all cancers.^[^ ^1^ ^]^ In China, there were an estimated 592 232 new cases of CRC and 30 9114 related deaths in 2022, accounting for ≈30% of the global cases of CRC during the same period.^[^ ^1^, ^2^ ^]^ Moreover, the incidence and mortality rates of CRC have been continuously increasing in recent years due to improvements in living standards, the aging of the global population, and sociodemographic changes.^[^ ^3^ ^]^ Fortunately, the 5‐year survival rate for CRC can be greatly increased from 15% to 91% if cancerous lesions are detected in the early stage, as the progression from precancerous lesions to invasive CRC is slow.^[^ ^4^ ^]^ CRC has an intervention window period of 3–10 years, making it the malignant tumor that is most suitable for screening. Research on large‐scale screening efforts in the United States has shown that using colonoscopy to screen for CRC reduced the incidence of CRC from 56 per 100 000 to 37 per 100 000 and reduced the mortality of CRC from 20 per 100 000 to 13 per 100 000 between the years of 2000 and 2018.^[^ ^5^ ^]^

China has implemented a population‐based colorectal cancer screening program in urban and rural areas with a high prevalence of CRC in the past decade. In contrast with developed countries such as the United States, China has a large population, an insufficient number of endoscopists, and a large gap in the quality of primary health care; therefore, the screening guidelines for CRC in China are based on a preliminary screening of individuals in the appropriate age group, followed by colonoscopy for high‐risk groups to achieve the best health economic effects.^[^ ^6^ ^]^ Currently, the most common preliminary screening strategy is the use of a high‐risk questionnaire combined with fecal occult blood tests (FOBTs), such as guaiac‐based FOBTs (gFOBTs) or fecal immunochemical tests (FITs).^[^ ^7^ ^]^ FOBTs are the most widely used noninvasive stool‐based test for CRC screening and have a higher adherence rate than colonoscopy because of their convenience and relatively low costs. However, FOBTs have limited sensitivity, as the FITs alone detect only 36.7% of advanced adenomas (AAs),^[^ ^8^ ^]^ which leads to missed detection of early‐stage colorectal cancer and AA. Moreover, the limited specificity of FITs results in a relatively high false‐positive rate and leads to a waste of colonoscopy resources. For example, in the population‐based CRC screening program, the false‐positive rate of FITs reached 77.25%, resulting in many unnecessary colonoscopy examinations, as patients with positive results would require colonoscopy to verify the etiology of potential bleeding.^[^ ^9^, ^10^ ^]^ Colonoscopy is regarded as the gold standard for CRC screening and allows the simultaneous detection and removal of adenomas. However, the acceptance of screening via colonoscopy remains very low due to the invasive nature of the procedure and the requirement for extensive bowel preparation, which can cause discomfort or disgust. Even among high‐risk populations, the participation rate in screening colonoscopies is only 14%.^[^ ^11^ ^]^ Accordingly, accurate preliminary screening is important for reducing the number of false‐positive results in patients who undergo unnecessary colonoscopy examinations and for improving colonoscopy compliance.

Abnormal DNA methylation is regarded as an early molecular event in tumorigenesis; this event has received an increasing amount of attention, and it has been widely used as a biomarker for early screening and diagnosis of tumors.^[^ ^12^, ^13^ ^]^ Emerging evidence suggests that DNA methylation markers, such as Septin9, SDC2, SFRP2, NDRG4, and TIPF1, can be used as diagnostic markers for CRC, with sensitivities ranging from 72.4%−93.3% and specificities ranging from 73%−95.2%in blood or stool samples.^[^ ^14^, ^15^, ^16^, ^17^, ^18^, ^19^ ^]^ DNA methylation tests in stool samples are more accurate than other CRC screening methods, as specimens directly sourced from intestinal exfoliated cells can clearly reflect the status of the intestinal mucosa, thereby significantly reducing the colonoscopy workload.^[^ ^20^, ^21^ ^]^ For example, the sensitivities of methylated SDC2 in blood and stool samples from patients with CRC (stage I) were 55% and 72.7%, respectively.^[^ ^22^, ^23^ ^]^ However, DNA methylation is only an epigenetic modification that does not change the base sequence. Therefore, current methylation detection methods, whether based on sequencing, molecular hybridization, or PCR platforms, all need to be used to convert the modification into a different base sequence or identify the modification via specific enzyme recognition. To date, methylation detection methods based on bisulfite conversion have become the main choice because of their high conversion efficiency, acceptable repeatability, and relatively mature technology.^[^ ^24^ ^]^ Unfortunately, the bisulfite conversion process is harsh and time‐consuming, thus considerably reducing the detection efficiency and increasing the requirement for experimental skills. Unfortunately, due to the lack of better alternative methods, almost all of the abovementioned DNA methylation detection methods depend on cumbersome bisulfite conversion operations, which are difficult to automate, and well‐trained professionals are required to conduct experiments to ensure the reliability of the results. However, as a screening method for identifying high‐risk populations before colonoscopy, the method should be easy to perform, affordable, and more suitable for grassroots application and widely promoted technology. Thus, it is necessary to develop new methods for population‐based CRC screening at the primary level.

Another noteworthy issue is the relationship between methylation locations (specific sites) and disease status. Aberrant DNA methylation at CpG sites in genes is one of the most common molecular alterations during tumorigenesis in all types of cancer.^[^ ^25^, ^26^, ^27^ ^]^ It is widely believed that a cluster of methylation sites, such as methylation haplotype blocks or a hypermethylated CpG‐rich region, is more relevant to disease.^[^ ^25^, ^28^, ^29^ ^]^ Interestingly, recent studies have shown that the weights related to diseases differ according to the specific site in the methylation site cluster.^[^ ^30^, ^31^ ^]^ A single methylation site could yield high sensitivity and specificity for detecting CRC and precancerous lesions.^[^ ^26^, ^32^ ^]^ However, existing non‐sequencing‐based methylation detection technologies, such as PCR, cannot detect the methylation status of specific sites; therefore, it is challenging to detect specific methylation sites based on PCR strategies that can meet the needs of daily clinical practice.

**Figure 1:**
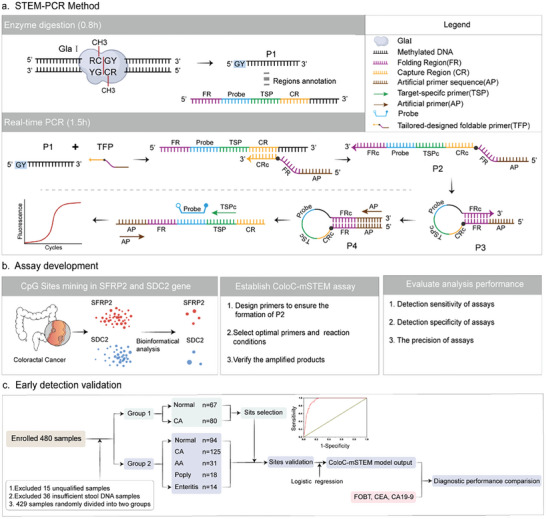
The scheme of early detection of CRC based on CpG sites detected by the ColoC‐mSTEM. a) STEM‐PCR method: GlaI specifically recognizes and cleaves the 5′‐R (5mC) GY‐3′/3′‐YG (5mC) R‐5′ DNA sequence, thus producing a specific 5′ terminal sequence (P1). A tailored‐designed foldable primer (TFP) that hybridizes with P1 and stops at the 5′ end resulting in the TFP extension products (P2) having the FRc sequence at the 3′end. Consequently, the FRc sequence in P2 spontaneously pairs with the FR sequence to generate a construct that can self‐fold (P3) and self‐prime into a complete hairpin structure (P4) without a 3′ end overhang. P4 can initiate PCR amplification, thus enabling the identification of the methylation status of a specific site. The lowercase letter c indicates the antisense of the sequence. b) Assay development. Bioinformatics methods were used to mine CRC‐related methylation sites in the *SDC2* and *SFRP2* genes in the TCGA database. Assays for identifying target methylation sites were developed based on the principle of STEM‐PCR technology, and the tolerance of the reaction system to clinical sample testing was verified. The sensitivity, specificity, accuracy, and precision of the assays were subsequently verified to ensure that they could be used for subsequent clinical sample testing. c) Validation of the early detection of ColoC‐mSTEM. Four hundred twenty‐nine of the enrolled samples qualified for further analysis. Group 1 was used for preliminary screening of CRC‐related methylation sites in stool DNA through cluster analysis and receiver operating characteristic (ROC) analysis. The methylation sites selected from Group 1 were detected in Group 2 samples to further confirm the clinical diagnostic performance and explore the early diagnostic ability of the identified sites in stool DNA. Finally, logistic regression analysis was conducted to establish a diagnostic model for CRC via the ColoC‐mSTEM, and the clinical diagnostic performance of the methylation sites and traditional detection indicators was compared.

Fortunately, the recently developed specific terminal‐mediated polymerase chain reaction (STEM‐PCR) technique is an innovative, bisulfite‐free method for site‐specific methylation detection that uses simple, low‐cost, and widely accessible PCR‐based workflows; furthermore, this technique has a sensitivity 20 times higher than that of current gold‐standard bisulfite conversion techniques.^[^ ^33^ ^]^ As shown in **Figure** **1**a, unlike bisulfite conversion, which recognizes DNA methylation via sequence differences between methylated and unmethylated templates generated after chemical conversion, STEM‐PCR uses methylation‐dependent restriction endonucleases (MDRE) to specifically recognize and cleave specific methylation sites, thereby generating templates with specific 5′ termini (P1). Although there are multiple MDRE available to recognize and cleave methylation sites to be suitable for STEM‐PCR strategy, the recognition sequence of GlaI covers ≈25% of the methylation sites, which can reduce fragmentation degradation caused by excessive cleavage of the template due to dense recognition sites, ensuring subsequent PCR effective amplification. In addition, unlike the other MDRE enzymes that produce sticky ends (such as MspJI), GlaI directly cleaves the phosphodiester bond between CG bases to produce blunt ends,^[^ ^34^ ^]^ which can reduce site competition caused by spatial hindrance. Therefore, GlaI is a high digestion efficiency MDRE enzyme for STEM‐PCR.

In the real‐time PCR stage, the tailor‐designed foldable primer (TFP) recognizes the P1 templates and triggers linear extension until reaching the 5′ end of P1, resulting in the TFP extension products (P2) having the Folding Region (FRc) sequence at 3′end. Consequently, the FRc sequence in P2 spontaneously pairs with the FR sequence, thus generating a self‐folding product (P3). Then, P3 self‐extends into a complete hairpin structure (P4) without 3′ end overhang, which can initiate the exponential amplification process using Artificial Primer (AP) and Target‐specific Primer (TSP) without requiring a time‐consuming bisulfite pretreatment process. Due to the cleavage of specific methylation sites, STEM‐PCR cleverly achieves precise identification of specific methylation sites on the PCR platform. Briefly, DNA methylation can be detected within 2.5 h by the STEM‐PCR strategy, which includes only enzyme digestion and real‐time PCR. This simple, economical, and efficient methylation detection method is an ideal technology for large‐scale application in primary health care. However, the STEM‐PCR method has not yet been validated in large‐scale samples for clinical diagnosis.

In the present study, we explore whether this method can be used as a clinically accessible new tool for disease early diagnosis and whether specific methylation sites can serve as tumor markers in colorectal cancer, which can benefit most from screening. As shown in Figure 1b, the innovative STEM‐PCR technique was used for the first time to discover and verify specific methylation sites in colorectal cancer through a case‒control study to address the dilemma of large‐scale CRC screening based on DNA methylation detection in clinical practice. Due to the focus on grassroots accessibility PCR platforms that can detect only a small number of CpG sites, selecting the most relevant CpG sites for CRC is another important challenge we need to address to accurately identify early‐stage CRC and even precancerous lesions. Thus, based on The Cancer Genome Atlas (TCGA) database, a self‐developed bioinformatics analysis was used to prescreen and filter colorectal cancer‐relevant methylation sites detected by the Illumina Infinium MethylationEPIC BeadChip (HM850K) in the *SFRP2* and *SDC2* genes, which were reported to have high sensitivity for detecting CRC in China.^[^ ^35^ ^]^ This analysis aimed to improve the efficiency of site selection. With the use of STEM‐PCR technology, the ColoC‐mSTEM assay was developed for each selected site, and the performance of the ColoC‐mSTEM assay was systematically evaluated to ensure the reliability of the established assays.

A case‒control study of 480 individuals was subsequently conducted to systematically evaluate the clinical performance of the selected methylation sites and further confirm the sites that can be used as biomarkers for early screening or diagnosis of colorectal cancer. Group 1 preliminarily verified the clinical relevance of the selected sites, while Group 2 further verified the diagnostic performance of the clinically related sites. Finally, several diagnostic models of colorectal cancer based on DNA methylation were established, and in‐depth comparisons were conducted between the screening and diagnostic performance of the FOBT and tumor markers (Figure 1c).

## 2 Results

### 2.1 The Data Mining Based on Machine Learning of Colorectal Cancer‐Associated Methylation of the *SFRP2* and *SDC2* Genes

**Figure 2:**
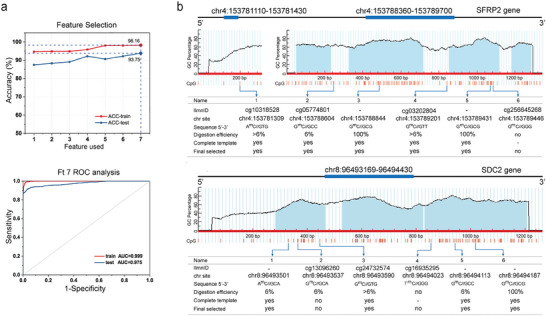
Locations selected for developing ColoC‐mSTEM assay. a) The accuracy of the selected loci for distinguishing CRC tissue from normal tissue (top) (1. SDC2‐4; 2. SDC2‐2; 3. SDC2‐3; 4. SFRP2‐6; 5. SFRP2‐1; 6. SFRP2‐2; 7. SFRP2‐4); For Ft7 model, a ROC curve was plotted for CRC versus normal tissues (below). b) The final selected locations for the ColoC‐mSTEM assay.

Existing evidence indicates that methylation of the *SDC2* and *SFRP2* genes is a valuable diagnostic marker for colorectal cancer; this finding has been validated in the Chinese population.^[^ ^19^, ^35^ ^]^ Therefore, herein, CRC‐related methylation sites were screened based on the *SFRP2* and *SDC2* genes to explore whether the specific methylated CpG sites could be used as diagnostic markers for CRC. Bioinformatic methods based on machine learning were used to screen CRC‐related methylation sites in the *SFRP2* and *SDC2* genes from the TCGA and Gene Expression Omnibus (GEO) databases. First, the t‐test was performed to compare CRC tumor tissue samples and normal tissue samples, and sites with *p‐*values < 0.05 were selected. Then, the F‐score feature selection algorithm^[^ ^36^ ^]^ combined with the coordinate distance of each site was used to select CpG sites with strong discrimination ability and relatively concentrated locations. The training set data, which included 299 colorectal tumor tissues and 298 paracancerous tissues of colorectal or other tumors from the TCGA database, were used to discover CRC‐related methylated CpG sites. Sixty‐four paracancerous tissues and 64 colorectal cancer tissues from the GEO database were used as the test set to validate the selected CpG sites. In the first round, 31 methylation sites in the *SDC2* gene and 42 methylation sites in the *SFRP2* gene with a *p*‐value < 0.05 for the t‐test were screened to examine their correlations with CRC in the TCGA database. While adding sites to construct an SVM classification model, the noise sites that reduce accuracy and the redundant sites that maintain accuracy were removed. Ultimately, 7 CpG sites were identified, and the single methylation site SDC2‐4:cg16935295 was the most effective at classifying CRC (the average methylation level of CpG sites in tissues was shown in Table S1, Supporting Information). As shown in **Figure** **2**a, the combination of 7 methylation sites (Ft7 model), which was based on the SVM model of SDC2‐4 and the addition of SDC2‐2 (cg13096260), SDC2‐3 (cg24732574), SFRP2‐6 (cg25645268), SFRP2‐1 (cg10318528), SFRP2‐2 (cg05774801), and SFRP2‐4 (cg03202804 sites), yielded a diagnostic accuracy of 98% (AUC = 0.99) for CRC in the training set and 93.7% (AUC = 0.97) in the test set. Therefore, we further analyzed whether the obtained methylated CpG sites in the Ft7 model could be digested by GlaI, a methyl‐directed DNA endonuclease that recognizes the 5′‐R (5mC) GY‐3′/3′‐YG (5mC) R‐5′ DNA sequence. Unfortunately, only 4 of the 7 methylation sites in the Ft 7 model could be digested by GlaI, and the digested products were used as complete templates for designing primers to establish a valid colorectal cancer DNA methylated STEM‒PCR assays (ColoC‐mSTEM) (Figure 2b). Due to the consensus of epigenetics, the possibility of co‐methylation occurring at adjacent CpG sites within a distance of 1000 bp,^[^ ^37^, ^38^ ^]^ the three sites that cannot be used to design ColoC‐mSTEM assays were replaced by adjacent sites no >100 bp away. The adjacent sites can be digested by GlaI and have complete templates for designing primers. (SFRP2‐5 (chr4:153789431) replaces SFRP2‐6, SDC2‐5 (chr8:96494113) replaces SDC2‐4, and SDC2‐1 (chr8:96493501) replaces SDC2‐2).

Additionally, the data were mined from the HM850K microarray, which does not cover all CpG sites. Therefore, we thoroughly studied the information about the sequences around the selected CpG sites and artificially supplemented SFRP2‐3 (chr4: 153788844) and SDC2‐6 (chr8: 96494187) to develop ColoC‐mSTEM based on the reported clinical data. The positions of the selected CpG sites in the *SFRP2* and *SDC2* genes are shown in Figure S1 (Supporting information). Specifically, the selected CpG sites in the *SDC2* gene were located mainly in the promoter and 5′UTR regions, while the distribution of methylation sites in the *SFRP2* gene was relatively wide, including in the 5′UTR, exon, 3′UTR, and noncoding region.

### 2.2 The Establishment of the Colorectal Cancer DNA Methylated STEM‒PCR (ColoC‐mSTEM) System and Key Factors Influencing Detection Performance

**Figure 3:**
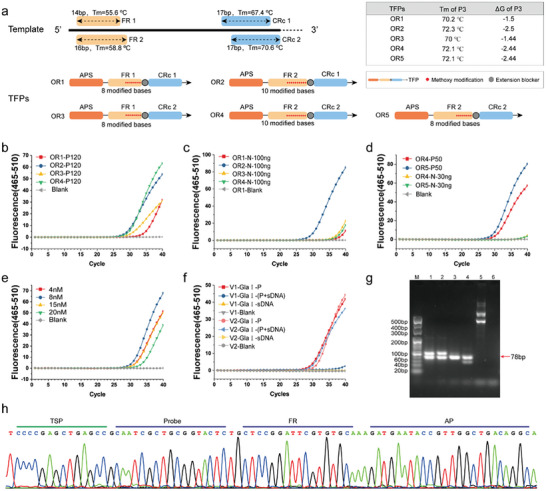
The process of establishing the ColoC‐mSTEM assay. a) TFP design for SDC2‐6. b) The effect of FR and CRc on the amplification efficiency of the positive template P120:120 copies of methylated DNA per test. c) The effect of FR and CRc on the specificity of negative template amplification; N‐100 ng: 100 ng of undigested DNA per test. d) Effect of methoxy modification on amplification performance; P50: 50 copies of methylated DNA per test; N‐30 ng: 30 ng of undigested DNA per test. e) Effect of TFP primer concentration on amplification performance. f) Comparison of the tolerances of different PCR systems for sDNA samples. GlaI‐P: GlaI‐digested 50 copies of methylated DNA per test; GlaI‐(P+sDNA): GlaI‐digested 50 copies of methylated DNA and 200 ng of sDNA; GlaI‐sDNA: GlaI‐digested 200 ng of sDNA. g) ColoC‐mSTEM PCR products separated by agarose gel electrophoresis line M:20 bp marker; line 1) 1000 copies per test; line 2) 100 copies per test; line 3) 40 copies per test; line 4) 10 copies per test; line 5) 10 ng undigested DNA per test. h) The sequence of ColoC‐mSTEM PCR products (78 bp of line 1 in F) detected by cloning sequencing.

It is crucial that the key influencing factors in the development of ColoC‐mSTEM include primer design, such as TFP, which includes regions of AP, FR, and Capture Region (CRc), and the optimization of PCR conditions. Here, taking the establishment of the SDC2‐6 ColoC‐mSTEM assay as an example, we first designed 5 types of TFPs with different FRs, CRc, and modified bases in the FR region, as shown in **Figure** **3**a. TFP is the determining primer for the formation of the stable hairpin structure (P3), which is the primary factor in the STEM‐PCR method. The Tm and Δ*G* of P3 mediated by TFP were then predicted at 66 °C by IDT to confirm whether the stem ring structure of P3 could be formed during PCR based on the design principles of our proposed STEM‐PCR method.^[^ ^33^ ^]^ Figure 3a shows that all five TFP primers can form stable P3 products with a Tm > 66 °C and a Δ*G* < 0. Moreover, in addition to the stable formation of the P3, it is also necessary to ensure that high concentrations of AP can open the hairpin structure (P4) to trigger subsequent PCR exponential amplification via STEM‐PCR. Therefore, the design of the FR region is equally important. As shown in Figure 3b, the amplification efficiency of OR2/OR4, which had 2 bases added to the FR, was better than that of OR1/OR3, indicating that the FR of TFP is a key factor for determining the efficiency of STEM‐PCR amplification. Because the CRc sequence is the region in which the TFP primer specifically recognizes P1 templates, the design of the CRc sequence is crucial for guaranteeing the specificity of the reaction system. Although OR2 and OR4 exhibited similar amplification efficiencies (Figure 3b), the OR2 system resulted in significant nonspecific amplification in nondigested genomes (100 ng), which lacked P1 templates. In contrast, no nonspecific amplification was detected in the OR4 system, as the Tm and sequence of CRc2 were different from those of CRc1 (Figure 3c). These results suggest that optimizing the CRc is crucial for improving the specificity of the STEM‐PCR system.

To further improve the amplification efficiency of the reaction system, the modified bases in the FR sequence and TFP concentrations were explored systematically. Compared to that in OR4, the number of methoxy modification bases in OR5 was reduced from 10 to 8, with no changes in the FR sequence or length, Tm of the P3 or Δ*G* of the P3, further improving the amplification efficiency without triggering nonspecific amplification (Figure 3d). Moreover, the concentration of TFP also affected the amplification efficiency; for example, the 8 nm OR5/PCR combination had a better amplification efficiency, and lower (4 nm) or higher (15–20 nm) OR5 concentrations had an inhibitory effect on amplification (Figure 3e).

However, the PCR system is another important factor for ensuring the tolerance of clinical samples due to the large amount of PCR inhibitors in stool DNA (sDNA) samples. Although the performance of the V1 (0.5U Taq DNA Polymerase, 3.0 mm Mg^2+^) and V2 (1.0U Taq DNA Polymerase, 1.5 mm Mg^2+^) systems in pure genomic DNA amplification status is consistent, the V1 system cannot detect positive methylation signals when there are 50 copies of positive templates in 200 ng sDNA mixture; however, the V2 system can detect these signals (Figure 3f). These results suggest that a high concentration of Taq DNA Polymerase seems to improve the anti‐interference ability of the PCR system. Therefore, the V2 system, which is tolerant of complex sDNA, was selected for assay performance verification in subsequent experiments.

The final step in determining the ColoC‐mSTEM assay was to verify whether the amplified product fragment size and sequence were consistent with the expected target sequence. The agarose gel electrophoresis results showed that different concentrations of methylated template (1000‐100‐40‐10 copies per test) in the SDC2‐6 reaction system had obvious target amplification products at 78 bp, and there were fewer nonspecific amplification bands and no target products in the nondigested genomes (30 ng) per test (Figure 3g). The sequence of the target 78 bp amplification product was the same as the expected target sequence, including the TSP, probe, FR, and AP sequences (Figure 3h), demonstrating that the developed ColoC‐mSTEM can successfully achieve specific amplification of single‐methylated SDC2‐6. By optimizing the sequence, concentration of primer, and amplification program, a ColoC‐mSTEM system was ultimately established for the 9 candidate locations. The amplicon sequences and corresponding probes for detecting the other 8 selected CpG sites have been listed in Figure S3 (Supporting Information).

### 2.3 The Analysis of the Performance of ColoC‐mSTEM

In the early stage of tumor or precancerous lesion development, abnormal methylated DNA in intestinal exfoliated cells from stool DNA occurs at considerably low abundance with extremely high levels of background noise from unmethylated genomic DNA and bacterial DNA. Therefore, high‐sensitivity detection assays are particularly important when large amounts of interfering DNA are present. To be applicable to clinical research, the ColoC‐mSTEM was designed with β‐actin (ACTB) as an internal reference gene, fully methylated genomic DNA as the positive standard, and whole‐genome amplification products in which all cytosines in the sequence were unmethylated as the negative standard to study the performance of the established ColoC‐mSTEM assay.

**Figure 4:**
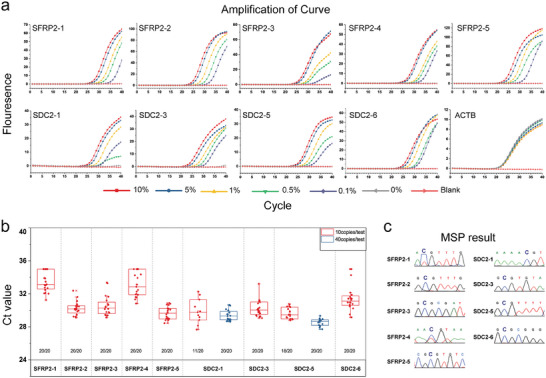
The sensitivity and accuracy of the ColoC‐mSTEM assay. a) The detection sensitivity of the SFRP2 locus in ColoC‐mSTEM assays (three replications). b) The results of 20 replicates of loci with 0.1% or 0.4% methylation template detected by ColoC‐mSTEM. c) A total of 10% of the methylation templates were detected by BSP.

To evaluate the analytical performance of the ColoC‐mSTEM assays, mixtures of 30 ng of genomic DNA (≈10 000 copies) with different methylation percentages of 10%, 5%, 1%, 0.5%, 0.1%, and 0% were detected. As shown in **Figure** **4**a, the ColoC‐mSTEM assays of 9 candidate locations in *SFRP2* and *SDC2* genes successfully detected 10%–0.1% methylation templates; moreover, no amplification of the 0% methylation template was detected, indicating that a 0.1% methylation signal under an unmethylated background was present. The ultimate sensitivity of ColoC‐mSTEM was confirmed by the fact that 20 replicates had a methylation ratio <0.1% for the 5 loci in the *SFRP2* gene and for the locations of SDC2‐3 and SDC2‐6. The other two candidate loci, SDC2‐1 and SDC2‐5, had a 100% detection rate with a 0.4% methylation ratio template (Figure 4b). Then, the 10% methylation template was further evaluated by bisulfite sequencing PCR (BSP), and the results revealed the presence of a methylated cytosine at all 9 candidate locations, confirming the accuracy of the established ColoC‐mSTEM (Figure 4c). Specifically, templates with different methylation ratios of SDC2‐6 were also measured by bisulfite sequencing PCR (BSP); 10%−0.5% of the methylation template was detected as methylated SDC2‐6, but 0.1% of the template failed (Figure S2, Supporting Information). Therefore, the sensitivity of ColoC‐mSTEM was greater than that of gold‐standard BSP techniques (0.1% vs 0.5%), making it more suitable for detecting samples with a low methylation ratio.

The precision of the ColoC‐mSTEM was evaluated to ensure the accuracy and stability of the assays, which is crucial for clinical sample testing. Fully methylated genomic DNA at 5 concentrations (640, 320, 160, 80, and 40 copies per test) was detected three times on different days, with 7 replicates per detection. The result shows that all 8 CpG sites except for SDC2‐5 had a detection rate of 100% (21/21) within 640‐40 copies per test and had a CV <5% with no amplification signal in the blank control (Tables S2 and S3, Supporting Information). These results indicate that the accuracy and precision of the 8 methylation sites were acceptable and stable enough for detecting clinical samples. SDC2‐5 was only acceptable when the concentration was >80 copies per test, although all 21 replications were detectable at 80 and 40 copies per test; however, they all had a CV > 5%.

### 2.4 Clinical Diagnostic Performance of the ColoC‐mSTEM for Identifying CRC in Tissues

To verify the correlation between the 9 selected CpG sites and CRC, we first investigated the relative methylation level of the CpG sites in tissue samples via ColoC‐mSTEM. Except for the negative correlation between the relative methylation level of SFRP2‐1 and colon cancer, the relative methylation levels of the other 8 CpG sites were positively correlated with colon cancer (Figure S4, Supporting Information). The relative expression levels of all the methylation sites in paracancerous tissues were significantly different from those in colorectal cancer tissues (*p <* 0.05), indicating that the selected methylation sites are highly useful diagnostic markers for colon cancer.

### 2.5 Clinical Diagnostic Performance of the ColoC‐mSTEM for Identifying CRC in Stool‐Derived DNA

As the exfoliation of tumor cells in stool occurs earlier than vascular invasion during colorectal carcinogenesis, the stool DNA test is more suitable for the early detection of CRC.^[^ ^20^ ^]^ To determine the clinical diagnostic performance of the selected locations by the ColoC‐mSTEM in terms of stool DNA, 480 participants were enrolled, and the percentage of qualified sample collection was 96% (465/480). Then, 36 stool DNA (sDNA) samples with insufficient human DNA (Ct value of ACTB > 24.5) were excluded, among which the human genome amount was less than 20 copies/PCR. Finally, 429 (92.2%) of 465 participants with sufficient human DNA were fully evaluated.

**Figure 5:**
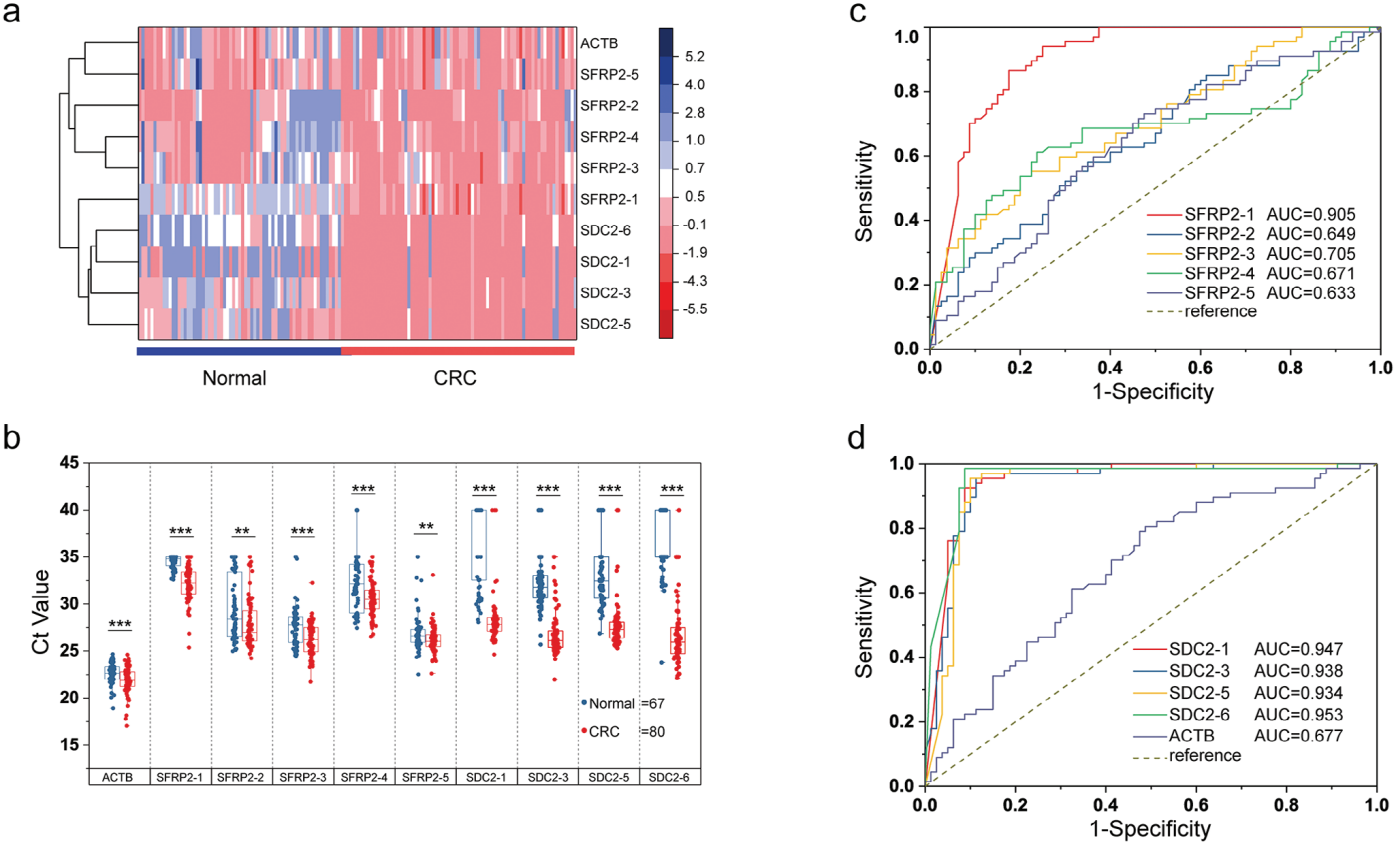
The clinical performance of the 9 selected locations. a) Unsupervised hierarchical clustering of methylation markers differentially methylated between 67 normal and 80 CRC subjects in group 1. Each column represents an individual patient, and each row represents a methylation marker. b) Comparison of Ct values between normal controls and CRC patients in the training group. c) Receiver operating characteristic (ROC) curves and area under the curve values (AUCs) of the 5 loci in the *SFRP2* gene. D) ROC curves and the AUCs of the 4 loci in the *SDC2* gene. ** *p* < 0.01, *** *p* < 0.001.

To screen and validate the colorectal cancer‐related methylation sites mentioned above, stool samples were divided into two groups. Group 1 preliminarily verified the clinical relevance of the selected CpG sites in sDNA, and the methylation levels of SFRP2‐1, SDC2‐1, SDC2‐3, SDC2‐5, and SDC2‐6 were different between normal individuals and CRC patients (**Figure** **5**a). However, the average Ct value of the 9 selected loci identified by the ColoC‐mSTEM in CRC sDNA sample was significantly lower than that in the normal group (Figure 5b). Unexpectedly, the methylation level of the SFRP2‐1 site in colorectal cancer tissue samples was relatively low, but its methylation level in the sDNA of CRC patients was actually greater than that in the normal group (*p <* 0.001). The methylation levels at other CpG sites in the sDNA samples were consistent with the results for the tissue specimens. To confirm the clinical performance of the 9 selected locations, the Ct value was further analyzed by examining the receiver operating characteristic (ROC) curve. As shown in Figure 5c, of the 5 methylation sites located in the *SFRP2* gene, only SFRP2‐1 could be used to distinguish CRC patients from normal individuals (AUC = 0.905). The area under the curve (AUC) for other locations was less than 0.75 (ranging from 0.633 to 0.705), indicating poor diagnostic performance. Notably, all the selected loci in the *SDC2* gene (SDC2‐1, SDC2‐3, SDC2‐5, and SDC2‐6) exhibited good performance in diagnosing CRC, with AUC values of 0.953, 0.938, 0.947, and 0.934, respectively (Figure 5d). Moreover, the AUC of ACTB was 0.677, suggesting that ACTB has no obvious clinical diagnostic value. Subsequently, for further clinical performance confirmation, only 5 of the 9 selected locations detected by ColoC‐mSTEM with a higher diagnostic power in stool DNA were selected.

**Figure 6:**
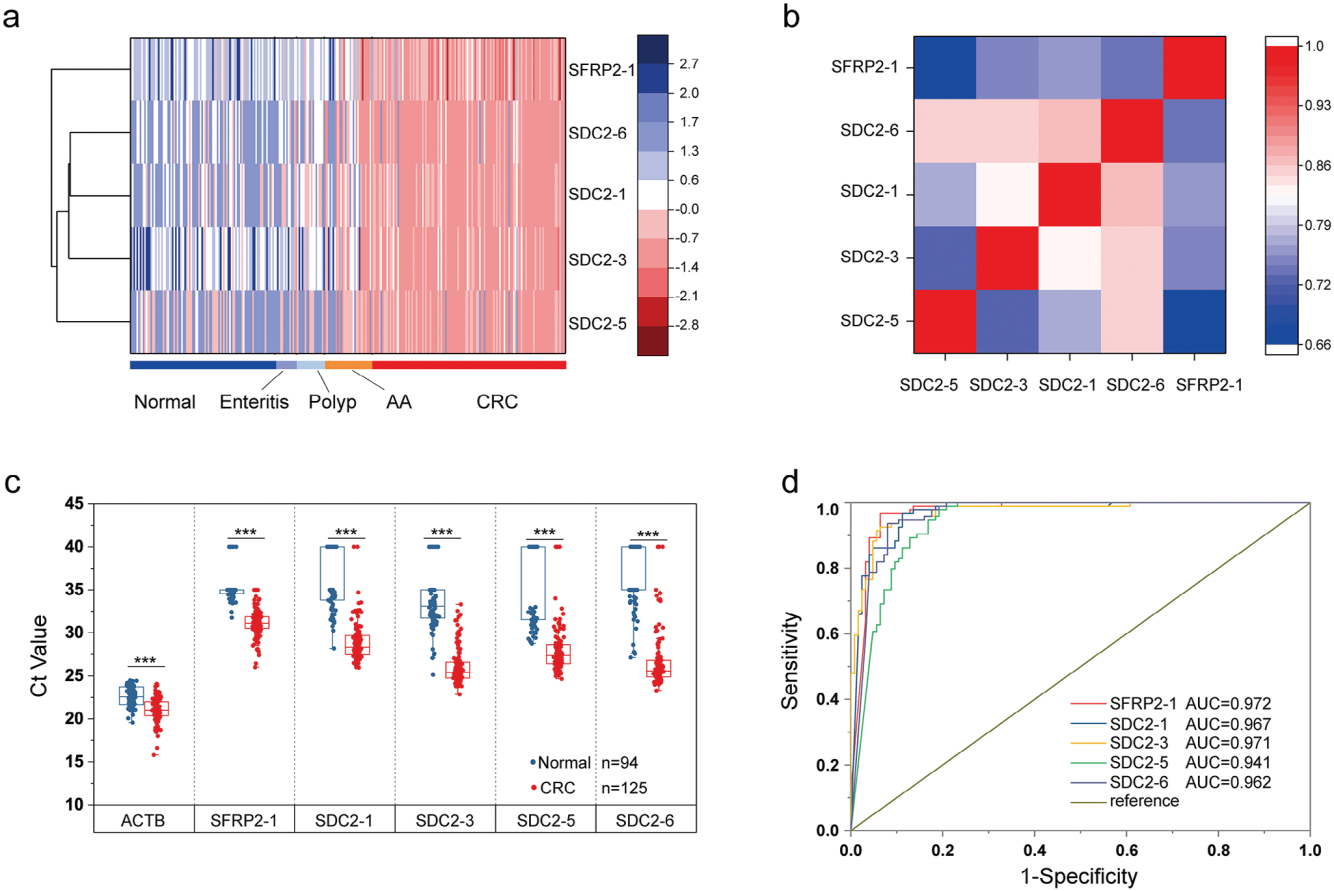
The clinical performance of the 5 selected markers was further confirmed. A) Unsupervised hierarchical clustering of methylation markers differentially methylated between 94 normal, 125 CRC, 31 AA, 18 polyps, and 14 enteritis patients in group 2. b) Correlation analysis of the markers. c) Comparison of Ct values between normal controls and CRC patients in group 2. d) ROC curves and the AUCs of 5 selected markers. *** *p* < 0.001.

The samples in Group 2 were used to further confirm the clinical diagnostic performance of SFRP2‐1, SDC2‐1, SDC2‐3 SDC2‐5, and SDC2‐6, which were selected from Group 1. As shown in **Figures** **6**a and 5 markers exhibited different methylation levels not only in CRC patients but also in advanced adenoma patients compared with normal individuals, while the methylation level was not obviously elevated in enteritis or polyp (<1.0 cm) samples. Further analysis of the correlations between the 5 selected markers was conducted. The results showed that SDC2‐6 was strongly correlated with SDC2‐1, SDC2‐3, and SDC2‐5, but the correlation between SDC2‐1, SDC2‐3, and SDC2‐5 was relatively low, suggesting that the detection of single SDC2‐6 by ColoC‐mSTEM could represent the other 3 locations in the SDC2 gene. A weak correlation was found between SFRP2‐1 and methylation markers in the *SDC2* gene (Figure 6b), demonstrating that SFRP2‐1 may be an independent diagnostic marker for CRC compared to the methylation markers in the *SDC2* gene. As shown in Figure 6c, the average Ct values of the 5 methylation markers were significantly lower in the CRC group (*n =* 125) than in the normal group (*n =* 94) (*p <* 0.05). The performance of the 5 methylation markers in identifying CRC was further confirmed by receiver operating characteristic (ROC) curve analysis, as the area under the curve (AUC) of all the markers was >0.94 (Figure 6d).

### 2.6 Clinical Diagnostic Model Based on the ColoC‐mSTEM Dataset

Unlike methylation‐specific PCR, which detects one region of methylated DNA using the bisulfite conversion strategy, the ColoC‐mSTEM assays established in the present study measure only one CpG site due to the unique characteristics of the STEM‐PCR method. Currently, there are almost no assays that can detect and quantify one CpG site on a PCR platform. To evaluate whether the specific CpG sites tested by the ColoC‐mSTEM could discriminate CRC patients from normal individuals, we analyzed the sensitivity and specificity of different CpG single‐site combinations and applied a multilocation model via logistic regression analysis of methylation markers (with the forward: conditional method). The diagnostic performance for CRC increased as the number of methylation sites increased, and the combination of SDC2‐6, SFRP2‐1, and SDC2‐1 in Model 3 showed the best diagnostic performance (AUC = 0.975) (Table S4, Supporting Information).

**Figure 7:**
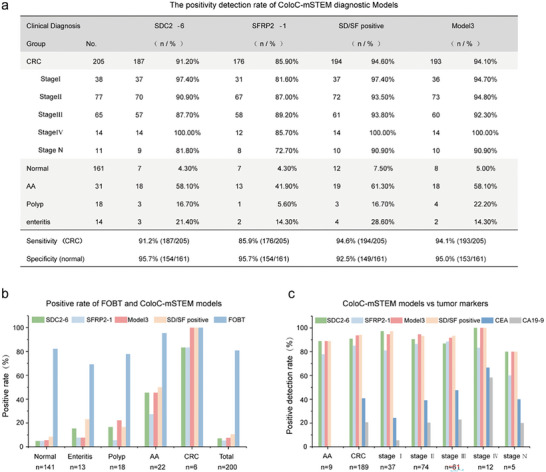
The effectiveness of ColoC‐mSTEM diagnostic Models. a) The positivity detection rate of ColoC‐mSTEM diagnostic Models in all samples. b) Comparison of the positivity rate between the FOBT and ColoC‐mSTEM methods. c) Comparison of the positivity rate between ColoC‐mSTEM and protein markers.

To analyze the effectiveness of ColoC‐mSTEM, Model 3, SDC2‐6, SFRP2‐1, and the combination of SDC2‐6 or SFRP2‐1 positive (named SD/SF positive) were treated as four different ColoC‐mSTEM diagnostic strategies. The cutoff values of Model 3, SDC2‐6, and SFRP2‐1 were determined using the Youden index. As shown in **Figure** **7**a, the sensitivities of SDC2‐6 and SFRP2‐1 for CRC detection were 91.2% and 85.9%, respectively, with a specificity of 95.7%. Notably, the combination of SFRP2‐1 and SDC2‐6 further improved the positivity rate of CRC at any stage. The sensitivities of Model 3 and the SD/SF‐positive combination for detecting CRC were 94.1% and 94.6%, respectively, with specificities of 95% and 92.5%, respectively. Except for the use of SFRP2‐1 alone, the detection rate for early‐stage CRC (stage I) reached 90%–97.4% for the other three methods. Importantly, four methylation diagnostic models also had high sensitivity for AA samples. The percentages of patients with AA‐positivity detected by SDC2‐6 and SFRP2‐1 were 58.1% and 41.9%, respectively, but the percentage of patients with AA‐positivity reached up to 61.3% when the two sites were combined (i.e., SD/SF‐positive). The detection rate of AA in Model 3 was consistent with that in SDC2‐6. The percentage of polyps and enteritis ranged from 5.6% to 28.6% when polyps were detected by the 4 ColoC‐mSTEM diagnostic models. Therefore, Model 3 had the best CRC diagnostic performance, and a single SDC2‐6 site had good diagnostic performance (91.2% sensitivity, 95.7% specificity).

### 2.7 Comparison of the Diagnostic Models and Other Noninvasive Screening Methods for ColoC‐mSTEM

We further compared the ColoC‐mSTEM to alternative noninvasive screening methods. A total of 200 individuals, FOBT positive and/ or high‐risk Questionnaire, were included in the screening group, including 141 normal controls, 22 individuals with AA, 6 individuals with Ca, 13 individuals with enteritis, and 18 individuals with polyps. Patients underwent FOBTs and stool DNA methylation tests. The false‐positive rate of the SDC2‐6, SFRP2‐1, Model 3, and SD/SF‐positive DNA methylation diagnostic models, i.e., the proportion of positive results detected in clinically normal or non‐adenomatous samples, are significantly lower than that of FOBT, especially for normal samples (4.95%, 4.96%, 5.67%, and 8.51%, respectively, vs 82.27%) (Figure 7b). In patients with AA, the positive rate detected by the ColoC‐mSTEM was lower than that detected by the FOBT (45.45%, 27.27%, 45.45%, and 50%, respectively, vs 95.45%). However, in patients with CRC, similar to those with FOBT, the positivity rates of Model 3 and SD/SF‐positive all reached 100%. More importantly, the specificity of the ColoC‐mSTEM for the non‐CRC population was significantly higher than that of the FOBT, indicating that the DNA methylation detection method has the potential to replace or supplement the FOBT as a primary screening tool for population‐based CRC screening to reduce the false‐positive rate of primary screening in normal people.

Moreover, 199 individuals, including 9 with AA (high‐grade adenoma or >1.5 cm) and 189 with CRC, were analyzed for carcinoembryonic antigen (CEA), carbohydrate antigen 19‐9 (CA19‐9), and ColoC‐mSTEM. As shown in Figure 7c, when CEA and CA19‐9 were used, no positive results were found in patients with AA, while SFRP1 yielded a positivity rate of 7/9 (77.9%) and SDC2‐6, Model 3 and SD/SF‐positive yielded positivity rates of 8/9 (88.89%). The performance of ColoC‐mSTEM was found to be superior to that of CEA and CA19‐9 across all CRC stages. The positivity rate reached 90% when using the SDC2‐6, Model 3, and SD/SF‐positive methods, while the positivity rates were lower for the CEA (40.74%) and CA19‐9 (20.63%) methods, especially for stage I patients (97.3%, 94.59%, 97.30%, 24.32%, and 5.41%). As expected, the sensitivity of ColoC‐mSTEM to the early stage of CRC (especially stage I‐II) and high‐grade AA was significantly higher than that of conventional clinical detection markers (CEA, CA19‐9). These results suggest that, compared with current conventional tumor protein markers, ColoC‐mSTEM is not only suitable for screening but also more effective for early diagnosis (auxiliary diagnosis).

## 3 Discussion

For the past decade, China has implemented a population‐based screening program in urban and rural areas with high prevalences of CRC to reduce the incidence and mortality of this tumor. Due to the large population base and the gaps in medical care between different regions, CRC screening programs have not been promoted nationwide. Additionally, under the screening guidelines for CRC in China, the relatively high false‐positive rate of primary screening methods combined with high‐risk questionnaires and FOBT results not only leads to a waste of colonoscopy resources but also reduces the rate of compliance for colonoscopy. To achieve the best health and economic effects, a more accurate primary screening method is required. Fortunately, the methods for preventing and controlling COVID‐19 in China have strengthened the ability of primary medical institutions to develop nucleic acid‐based molecular detection methods. Correspondingly, the largest battlefield for population‐based tumor screening is in primary medical institutions. Therefore, new screening methods with a low false‐positive rate, easy operation, low cost, and access to primary level for large‐scale promotion can lead to lower incidences of tumors.

In our current research, we established ColoC‐mSTEM assays for 9 specific CpG sites related to CRC using the innovative STEM‐PCR technique we developed, which can efficiently detect and quantify specific methylation sites via a simple, operation‐friendly, and low‐cost q‐PCR platform. Our results demonstrate that specific methylation sites do serve as effective tumor markers for CRC identification using the STEM‐PCR platform. Therefore, the utilization of tailor‐designed bisulfite‐free STEM‐PCR technology, characterized by its simple and accelerated handling procedure as well as high sensitivity and specificity, represents a novel and promising approach for the screening of colorectal cancer in a large population in primary medical institutions.

In this research, ColoC‐mSTEM was applied for prescreening of multiple CpG sites in stool DNA, thus validating the clinical practical applicability of the STEM‐PCR method. At present, the detection of methylation status at specific sites mainly depends on next‐generation sequencing (NGS), pyrosequencing, or HM450K/HM850K sequencing after bisulfite conversion, which involves more than ten tedious steps, and none of the above three methods can meet daily routine test requirements.^[^ ^24^, ^39^, ^40^ ^]^ ColoC‐mSTEM assays depending on STEM‐PCR technology without sulfite conversion not only is easy to perform but can also achieve a high sensitivity of a methylation detection ratio of 0.1% for specific methylation sites, which is similar to the sensitivity of next‐generation sequencing but higher than that of pyrosequencing (5%). When detecting CpG sites in sDNA samples, the ColoC‐mSTEM strategy requires only three steps—sDNA extraction, enzyme digestion, and real‐time PCR—which take less than 4 hours and include less than 20 minutes of manual operation. All the advantages of STEM‐PCR, such as high detection sensitivity, short operation time, low operational capability requirement, and complete compatibility with conventional PCR platforms, make it a convenient and ideal methylation detection technology that truly meets the needs of primary clinics.

However, not all target methylation sites can be detected using STEM‐PCR technology, due to the limitation of MDRE recognition sites and the sequence dependence of primer design. In this study, 2 out of 7 selected CpG sites could not be recognized by GlaI, and another CpG site has no complete P1 template for primer design after GlaI digestion, as other CpG sites in the P1 template could be recognized by GlaI. The recognition sequences of MspJI and FspEI respectively cover ≈75% and 50% of methylated sites in the 450K database. Consequently, selecting appropriate MDREs for target CpG sites can significantly enhance the site applicability of STEM‐PCR technology.^[^ ^33^ ^]^ Furthermore, the design of primers targeting the CR and FR regions is pivotal for the efficacy of STEM‐PCR reagents and poses significant challenges. Developing suitable primer design software will help promote the application of STEM‐PCR technology.

In the present study, to address the intriguing and important clinical question of whether specific methylation sites can serve as effective tumor markers, the F score feature selection algorithm and SVM classification model were used to select the least specific methylation sites with the best colon cancer classification effect to establish ColoC‐mSTEM assays. Five selected methylation sites located in the promoter and 5′UTR regions of the genes were found to be useful for the diagnosis of colorectal cancer (AUC > 0.9). In particular, SDC2‐6 alone had a sensitivity of 91.2%, 93% (107/115), and 58.1% for detecting all‐stage CRC, early‐stage CRC (stage I–II) and AA, respectively, with a specificity of 95.7%. These results indicate that the specific CpG site (SDC2‐6, chr8: 96494187) in stool DNA could be a powerful diagnostic marker for the early diagnosis of colorectal cancer. Most notably, the clinical performance of a single SDC2‐6 locus for early‐stage CRC was even superior to the reported performance of MSP in detecting *SDC2* gene methylation (Table S5, Supporting Information).^[^ ^19^, ^41^, ^42^ ^]^ For example, Oh TJ. et al. reported that the sensitivity of fecal DNA SDC2 methylation in patients with colorectal cancer was 90.0%, whereas that in patients with early‐stage CRC was 83.3% (stage I), with a specificity of 90.9%.^[^ ^41^ ^]^ A multicenter clinical study by Wang JP et al. reported that the sensitivity of SDC2 methylation was 83.8% for CRC and 87% for early‐stage CRC (stage I‐II), with a specificity of 98%.^[^ ^19^ ^]^ The detection of *SDC2* gene methylation in the above studies was based on the MSP method after bisulfite conversion, which usually measures the methylation level of regions containing multiple CpG sites covered by methylation‐specific primers and probes. In our previous study, there was a significant difference in methylation between SDC2‐3 and SDC2‐6, although the distance between the two CpG sites was <600 bp (the NGS data are shown in Table S6, Supporting Information). On the other hand, not all methylation sites in detection regions have clinical diagnostic significance, and the diagnostic performance of methylation sites is related to different functional domains of the genome.^[^ ^25^, ^30^ ^]^ Our results showed that the clinical diagnostic performance of the methylation site in the 5′UTR (SFRP2‐1, AUC = 0.905) was superior to that of the other sites in exons (SFRP2‐2, AUC = 0.649), 3′UTRs (SFRP2‐3, AUC = 0.705) or noncoding regions (SFRP2‐4, AUC = 0.671 and SFRP2‐5, AUC = 0.633). Even within the same region, the clinical diagnostic performance of SDC2‐5 (AUC = 0.934) and SDC2‐6 (AUC = 0.953) also varied, indicating that the detection of specific methylation sites may be superior to the detection of methylation regions because it can effectively shield against interference from other methylation sites within the region. The results of the present study indicate that specific methylation sites can serve as tumor diagnostic markers, and the development of STEM‐PCR technology could significantly promote the clinical translation of specific CpG sites as tumor diagnostic markers.

Furthermore, we also found that the presence of a single SDC2‐6 site in stool samples was more accurate than the presence of the specific site cg10673833 (sensitivity of 89.7% and specificity of 86.8%) in cfDNA samples.^[^ ^26^ ^]^ The markers are released into the stool via exfoliation during the progressive phases of tumorigenesis, which occurs at comparable rates between large precancers and all stages of cancer. However, these markers are released into the blood via vascular invasion, and their levels increase as the tumor progresses.^[^ ^20^ ^]^ Therefore, the selection of methylation sites and detection sample types are key factors affecting the clinical diagnostic performance of biomarkers. Moreover, the validation results of clinical samples in this study demonstrated that screening methylation sites by an algorithm based on machine learning is indeed effective and that this innovative algorithm has great clinical application value.

In population‐based CRC screening, primary screening‐positive subjects are recommended for colonoscopy. Reducing the false‐positive rate and enhancing the sensitivity for AA are the most difficult challenges in developing new primary screening methods. The methylation of SFRP2, BMP3, NDRG4, and TFPI2 has been reported to be useful diagnostic markers for CRC, and multitarget stool tests are usually used to improve the diagnostic performance of CRC.^[^ ^43^, ^44^, ^45^, ^46^ ^]^ The sensitivities were 86.2%–93.4% for CRC and 46.2%–61.5%for AA, with the specificity ranging from 89.1%– 94.3% in the published research (Table S5, Supporting Information).^[^ ^8^, ^17^, ^18^ ^]^ Like in these previous studies, we also found that SFRP2‐1, when combined with SDC2‐6, improved the diagnostic sensitivity. The sensitivity of SD/SF‐ positive for detecting CRC and AA increased to 94.6% and 61.8%, respectively, but the specificity slightly decreased to 92.5%. Model 3 improved the sensitivity for detecting CRC to 94.1%, with nearly no decrease in the specificity. As expected, according to our multitarget analysis, the *SDC2* gene or SDC2‐6 methylation site was always the most common factor due to the good clinical diagnostic performance of *SDC2* gene methylation, especially at the SDC2‐6 methylation site. Other genes or methylation sites can assist in improving the sensitivity of patients to colorectal cancer. Notably, there is a difference in the methylation level of SFRP2‐1 site between exfoliated cells and epithelial tissues in the normal group in this study. We speculate that this phenomenon may be associated with epithelial cell apoptosis, such as Anoikis and Pyroptotic.^[^ ^47^ ^]^ Further research is necessary to elucidate these inconsistent results.

Despite the slightly lower specificity of FOBT in CRC compared to FIT, economic constraints lead to the predominant use of FOBT for large‐scale colon cancer screening in most regions of China,^[^ ^11^ ^]^ with FIT being employed only in a few economically developed areas for CRC screening.^[^ ^48^ ^]^ Encouragingly, ColoC‐mSTEM diagnostic models established in this study not only have a lower false positivity rate for noncancer population than FOBT but also have higher sensitivity than CEA and CA19‐9 for the discrimination of CRC patients, especially high‐grade or large (>1.5 cm) AA and early colorectal cancer. Therefore, the utilization of the ColoC‐mSTEM, a simple and operation‐friendly method, is a highly promising approach for population‐based CRC screening.

This study has limitations, as it involved small‐scale retrospective clinical research of patients and normal individuals, and multicenter prospective studies are needed for intensive evaluation of CRC screening via the ColoC‐mSTEM. This research has high requirements for the human gene content and purity of sDNA, so it is necessary to further improve the robustness of the reaction system. This study has not yet systematically investigated the effects of enzymes, reagent stability, PCR analysis mode, etc., the detailed research is needed in the future. In addition, the comparison between ColoC‐mSTEM and traditional methods was insufficient. However, the exact application of the ColoC‐mSTEM in large‐scale CRC screening, whether as a replacement for traditional primary screening or as a follow‐up step after the primary screening, remains a subject of inquiry. Further prospective research in real‐world settings is necessary to identify the optimal strategy for CRC screening.

## 4 Conclusions

This is the first study to develop a site‐specific methylation detection strategy using stool samples for the early detection of CRC based on bisulfite‐free STEM‐PCR technology. This method is characterized by high sensitivity, high accuracy, and easy operation, and it perfectly suits the needs of primary medical screening. Using ColoC‐mSTEM to detect the specific methylation site SDC2‐6, either alone or in combination with SFRP2‐1, had high diagnostic values for the detection of CRC and AA. Model 3 had a specificity of 95.0% and a sensitivity of 94.1% and 58.1% for detecting AA and CRC, respectively. Thus, the use of ColoC‐mSTEM may serve as a robust approach for the early detection of CRC, especially in the context of population‐based screenings in primary medical institutions. We believe that employing ColoC‐mSTEM as a follow‐up step after current primary screening methods could not only reduce the high false‐positive rates but also efficiently enhance the sensitivity of early CRC detection and cancer screening, thus maximizing the cost‐effectiveness and public health benefits of primary‐level CRC screening.

## 5 Experimental Section

### Study Design

The main contents of this study were as follows: 1. Mining and selecting CRC‐related methylation sites through bioinformatics analysis and constructing a ColoC‐mSTEM detection system. 2. A retrospective case‐control study was designed to evaluate the clinical diagnostic performance of stool DNA‐based pre‐selected methylation sites detected by ColoC‐mSTEM.

### Preliminary Mining of CRC‐Related Methylation Sites, Clinical Specimens

CRC‐related methylation sites were mined and selected from *SFRP2* and *SDC2* genes, which were reported to have a high sensitivity for detecting CRC in China.^[^ ^35^ ^]^ In TCGA database, 299 cases of CRC samples and 298 cases of normal samples (from the paracancerous tissue of 38 CRC and 260 other tumor patients) were used as the training set to screen CRC‐related methylation sites and establish the model. The test set, 64 cases of CRC and 64 cases of normal (from the paracancerous tissue of 41 CRC and 23 other tumor patients) in the GEO database was used for model verification. The mining criteria were as follows: 1. Calculate the *p* < value of *t*‐test between cancer samples and normal samples, and select sites with *p* < 0.05. Next, use the F‐score Feature selection algorithm to calculate the importance score of each site to represent its discrimination ability.^[^ ^36^ ^]^ At the same time, combine the coordinate distance of each site to select sites with strong discrimination ability and relatively concentrated locations. Add sites, in turn, to construct an SVM classification model and eliminate the noise sites that reduce accuracy or redundant sites that maintain accuracy. The training set was sampled 10 times repeatedly for 5‐fold cross‐validation, 80% of the data was selected each time. The GEO test set was fixed and tested independently. ColoC‐mSTEM assay was designed for the obtained locis above that could be digested by GlaI which specifically cleaves 5 ‘‐ R (5mC) GY‐3′/3‘‐ YG (5mC) R‐5′ DNA sequence with high digestion efficiency,^[^ ^34^ ^]^ and the fragment length after digestion was sufficient for primers design.

The target population enrolled comes from the Key Population Colorectal Cancer Screening Project in Zhejiang province (Linhai City) and CRC patients come from Renji Hospital, including patients with diagnosis of CRC, advanced adenoma (AA), polyps, enteritis, and normal controls (no abnormality showed in colonoscopy), based on the results of complete colonoscopy and pathology outcome.^[^ ^49^, ^50^ ^]^ We excluded participants who had a previous history of CRC, any chemotherapy or radiotherapy, incomplete information, or insufficient human DNA. The subjects were divided into two groups, group 1 : 67 normal and 80 CRC individuals were randomly selected as the initial screening training set to pre‐screen CRC‐related methylation sites. Group2: the remaining 94 normal individuals, 125 CRC individuals, 31 AA (≥1.0 cm adenoma, containing villous components or high‐grade intraepithelial neoplasia) individuals, 14 enteritis individuals, and 18 polyp individuals were used as validation sets to evaluate the clinical diagnostic performance of pre‐screened methylation sites from Group1, and further explored the performance of CRC diagnostic models based on methylation sites (Figure 1c).

The detailed information of subjects is shown in Table S7 (Supporting Information). All participants signed informed consent after being told the study details. The clinical study was approved by the ethics committee at each participating hospital and funded by the STAR project of SJTU. (Shanghai Jiaotong University School of Medicine, Renji Hospital Ethics Committee Approval letter, KY2020‐107; The Ethics Committee for Human Related Scientific and Technological Research at Shanghai Jiao Tong University, 2022262I)

### Stool Collection and DNA Extraction

Stool samples (2 g), avoiding blood, urine, and mucus were collected into 10 mL preservation solution before colonoscopy/ bowel preparation. The main components of the preservation solution were EDTA, Tris, NaCl, SDS, DMSO, and absolute alcohol. Stool samples with a collection amount <2 g were considered unqualified. Before stool DNA (sDNA) extraction, the samples could be stored at 4 °C (< 7 days) or at −80 °C for long‐term storage. ColoC‐mSTEM was performed on the samples in a double‐blind manner. Colonoscopy/pathological examination results were used as the gold standard for sensitivity and specificity analysis.

Samples containing preservation solution were mixed at 2500× rpm for 20 min, centrifugation at 4000× g for 10 min, then transferred 400 µL of supernatant to a new 1.5 ml centrifuge tube, centrifuged at 10 000× g for 3 min. The supernatants were removed and added with 20 µL precipitation solution A, 100 µL precipitation solution B, and 30 µL protease K, then mixed thoroughly. After 10 min of incubation at 65 °C, the mixture was centrifuged at 13 000× g for 3 min. the supernatants were removed, and magnetic beads were used to adsorb nucleic acids after adding the binding solution. The beads were washed with 80% ethanol and dried. The DNA samples were eluted by 120 µL of TE buffer and stored at −20 °C. Before carrying out ColoC‐mSTEM detection, sDNA was diluted 5 times with nuclear‐free water to avoid the inhibition of enzyme digestion and PCR. Due to the presence of a large number of intestinal bacteria in stool samples, the DNA of intestinal exfoliated cells only accounts for 0.01% of the total stool DNA. The content of human DNA in sDNA was detected using the self‐developed β‐actin gene (ACTB) q‐PCR reagent, and a Ct value of ACTB >24.5 indicates insufficient human DNA (less than 20 copies/ PCR reaction). The products of preservation solution and stool DNA extraction were provided by Shanghai Healzone Biotechnology Co, LTD.

### Analytic Performance of *SFRP2* Gene and *SDC2* Gene of ColoC‐mSTEM

Templates with different methylation ratios were prepared to verify the sensitivity and specificity of the developed ColoC‐mSTEM. Different amounts (1000, 500, 100, 50, 40, 10, and 0 copies) of fully methylated human genomic DNA (CpG methylated jurkat Genomic DNA, Thermos Scientific) were diluted into 30 ng (≈10 000 copies) of unmethylated genomic DNA to create mixtures with methylation percentages of 10%, 5%, 1%, 0.5%, 0.4%, 0.1% and 0%. To confirm the ultimate sensitivity of ColoC‐mSTEM, 0.1% or 0.4% concentrations were measured 20 times, and a 95% detection rate was considered an achievable sensitivity for reagents. No amplification in 0% templates (or CT value not less than those of 0.1% templates) was considered indicative of good specificity performance. The unmethylated genomic DNA was prepared by whole genome amplification of human genomic DNA using the Illustra GenomiPhi V2 DNA Amplification kit (QIAGEN).^[^ ^41^ ^]^ Additionally, the region lacking GlaI digestion sites of β‐actin gene was used as an internal reference to estimate the amount of amplifiable template. The replicability of STEM‐PCR reagents was verified with different concentrations of fully methylated templates (640, 320, 160, 80, 40 copies per test). ColoC‐mSTEM was performed in 21 replicates (7repliccates in 3 independent runs using the same real‐time PCR instrument).

### Digital PCR

Crystal digital PCR reactions were prepared using Perfecta multiplex qPCR ToughMix (Quanta Biosciences, USA), 1 µm primer, 0.25 µm probe, and 2 µL DNA template. 100 nm reference dye was added to allow adequate imaging of all droplets for analysis. To prepare Sapphire chips, 25 µL of PCR mix were pipetted into the inlet ports before the pressure‐permeable caps (Stilla Technologies) were positioned onto the loaded ports. The cycling conditions of the digital PCR were: 95 °C for 10 minutes, followed by 45 cycles of 95 °C for 30 s, and 60 °C for 30 s.

Image acquisition was performed with Naica Prism 4 reader with the following exposure times: blue channel: 100 ms; green channel: 70 ms; red channel 50 ms. The total number and quality control of droplets were calculated using reference dye (blue channel). The positive droplets were calculated through the green channel. The concentration of the template was precisely analyzed using Stilla Crystal Miner v2.4.0.3 automatically.

### ColoC‐mSTEM Measurement Based on STEM‐PCR Strategy

For measurement of methylated locates in SDC2 and SFRP2 gene, the target template was amplified by ColoC‐mSTEM after MDREs digestion. 4 µL sDNA or certain concentration genomic DNA was incubated with 6 µL MDRE mix including 12.5 U G1a I (SibEnzyme, Russian) and 1X Taq buffer. The MDRE reaction system was incubated at 30 °C for 30 min and inactivated at 65 °C for 20 min, then stored at 4 °C. The MDRE‐digested product was either used in the following Real‐time PCR as a template or stored at −20 °C for further use.

A total of 20 µL reaction mixture contained 200 nm universal primer, 200 nm terminal specific primer (TSP), 10 nm TFP primer, 200 nm Taqman probe, and 0.3 mm dNTP (containing dUTP), V2 PCR system (1X PCR buffer, 1U Champagne Taq DNA Polymerase, and 1U UDG, all purchased from Vazyme Biotech, China) or V1 PCR system (1X PCR buffer, 1.5 mm MgCl_2_, 0.5U Champagne Taq DNA Polymerase and1U UDG, all purchased from Vazyme Biotech, China) and 8 µL MDRE‐digested product. Real‐time PCR was performed on a LightCycler instrument 480II (Roche, Basel, Switzerland). Thermal cycling conditions were as follows: 37 °C for 5 min and then heated at 95 °C for 5 min, 10 cycles of 95 °C for 10 s, 66 °C (Tm1) for 90 s, then 40 cycles of 95 °C for 10 s, 65 °C (Tm2) for 30 s (acquisition the fluorescence signal at Tm2). Tm1 and Tm2 were separately dependent on the TFP primer and terminal‐specific primer. For each run, fully methylated genomic DNA (160 copies per test) was used as positive control, including no‐template control. Cycle threshold (Ct) value was calculated using Abs Quant/ 2nd Derivative Max method in Light Cycler 480 software. The test result was accepted only when the Ct value of ACTB was ≤24.5.

The clinical performance of pre‐screened locates was analyzed by the receiver operating characteristics curve (ROC) analysis. The cutoff value of Ct in ColoC‐mSTEM for each locates methylation was chosen based on ROC analysis on assay results. According to the cutoff value, the sensitivity and specificity of methylation sites in distinguishing CRC and normal subjects were calculated. Logistic regression analysis was used to explore the specific sites methylation diagnostic model of colorectal cancer.

### Bisulfite‐Based Sequencing

30 ng Genomic DNA with different methylation ratios was chemically converted unmethylated cytosine to uracil by sodium bisulfite, while the methylated cytosine was kept unchanged using EZ DNA Mehtylation‐Gold kit (Zymo Research, USA) according to manufacturer's instruction. Bisulfite‐converted DNA was either used immediately for methylation analysis or stored at −20 °C until further use. Bisulfite‐converted DNA was first amplified using nest amplification as follows: the reaction included 1×Uc Buffer for Library Amplification, 0.2 mm dNTP mix, 400 nm primer (primer F1 and R1 added in the first round, primer F2 and R2 added in the second round), and 1U Phanta Uc Super‐Fidelity DNA polymerase. The reaction was carried out on the ProFlex 3×32‐well PCR System Applied Biosystems with the following program: initial denaturation at 95 °C for 5 min; 10 cycles of touchdown PCR as 95 °C for 30 s, 65 °C for 30 s (−0.5 °C per cycle), 72 °C for 20 s; 25 cycles of 95 °C for 30 s, 55 °C for 30 s, 72 °C for 20 s; then incubation at 72 °C for 7 min. The first round of amplificons was diluted 100 times and served as template for nest methylated specific‐PCR (MSP). The methylated specific‐PCR products were measured using Sanger sequencing.

### Protein Detection

Fecal occult blood tests (FOBTs) were performed on fresh stool collected from Linhai Hospital using the One Step Fecal Occult Blood Test (colloidal gold) (Abon Biopharm (Hangzhou) Co., Ltd.). The positive threshold for the occult blood detection was a hemoglobin concentration of 100 ng mL^−1^. FOBT was performed twice, and any positive was considered positive. The levels of CA19‐9 and CEA levels in serum from patients collected from Renji Hospital were detected with electrochemiluminescence (Roach, Elecsys10). The normal reference values used for the two different biomarkers were as follows: CEA ≤ 4.7 ng mL^−1^ and CA19‐9 ≤ 27 U mL^−1^.

### Statistical Analysis

The data were presented as means or as values. The Ct values detected in tissue and stool DNA samples underwent the Kolmogorov‐Smirnov test to assess normality. The two‐tailed t‐test was used to analyze the difference in Ct values between normal and colorectal cancer groups for datasets adhering to a normal distribution. For datasets deviating from normality, the Mann–Whitney U test (two‐sided testing) was employed to analyze the significance of differences. ROC curves were constructed to evaluate the diagnostic performance of each methylated locus, and 95% confidence intervals (CI) were calculated. The cutoff value was determined using Youden's index. All the statistical analyses were conducted with IBM SPSS Statistic 26 and origin 2017. *p*‐value < 0.05 was considered statistically significant.

## Conflict of Interest

The authors declare no conflict of interest.

## Supporting information

Supporting Information

## Data Availability

The data that support the findings of this study are available from the corresponding author upon reasonable request.
