# Supplementary material for: Early Diagnosis of Colorectal Cancer Based on Bisulfite‐free Site‐specific Methylation Identification PCR Strategy: High‐Sensitivity, Accuracy, and Primary Medical Accessibility
Source: Adv Sci (Weinh). 2024 Jun 13;11(33):2401137. doi: 10.1002/advs.202401137 (PMC11434020; doi:10.1002/advs.202401137)
Supplement: Supplementary file 1 — Supporting Information [file ADVS-11-2401137-s001.docx]

Supporting information

**Early Diagnosis of Colorectal Cancer Based on Bisulfite-free Site-specific Methylation Identification PCR Strategy: High-Sensitivity, Accuracy, and Primary Medical Accessibility**

*Linqing Zhen^#^, Xinlu Tang^#^, Zhengguo Xu^#^, Yizhou Huang, Xiaohua Qian, Haiping Lin, Chao Li, Rong Cui, Hongsheng Fang, Hao Yang, Jiani Qiu, Zhaoqi Fang, Xiaohuan Peng, Yifeng Jin, Jianing Nie, Shiwei Guo, Yuguang Wang, Ming Zhong*, Hongchen Gu*, Hong Xu**


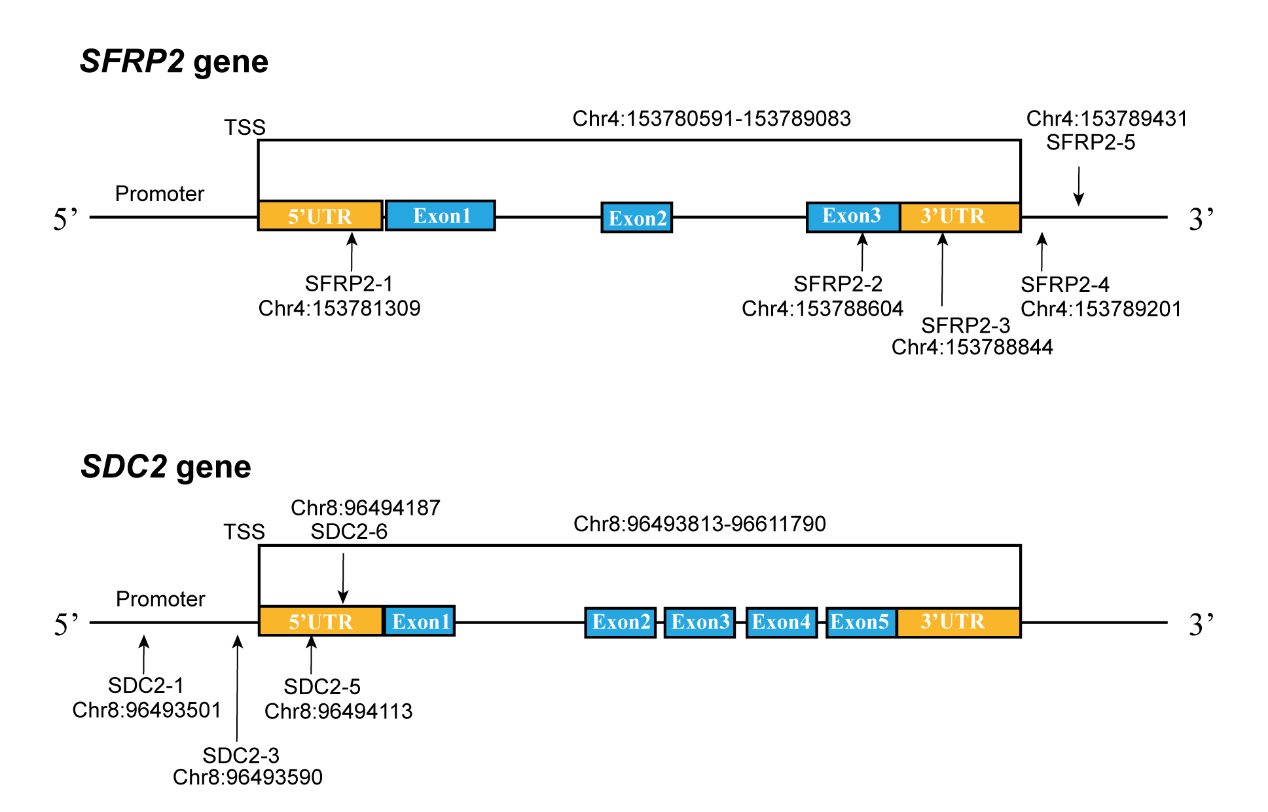


**Figure S1**. The position of selected methylation sites in the functional region of *SFRP2* and *SDC2* gene.


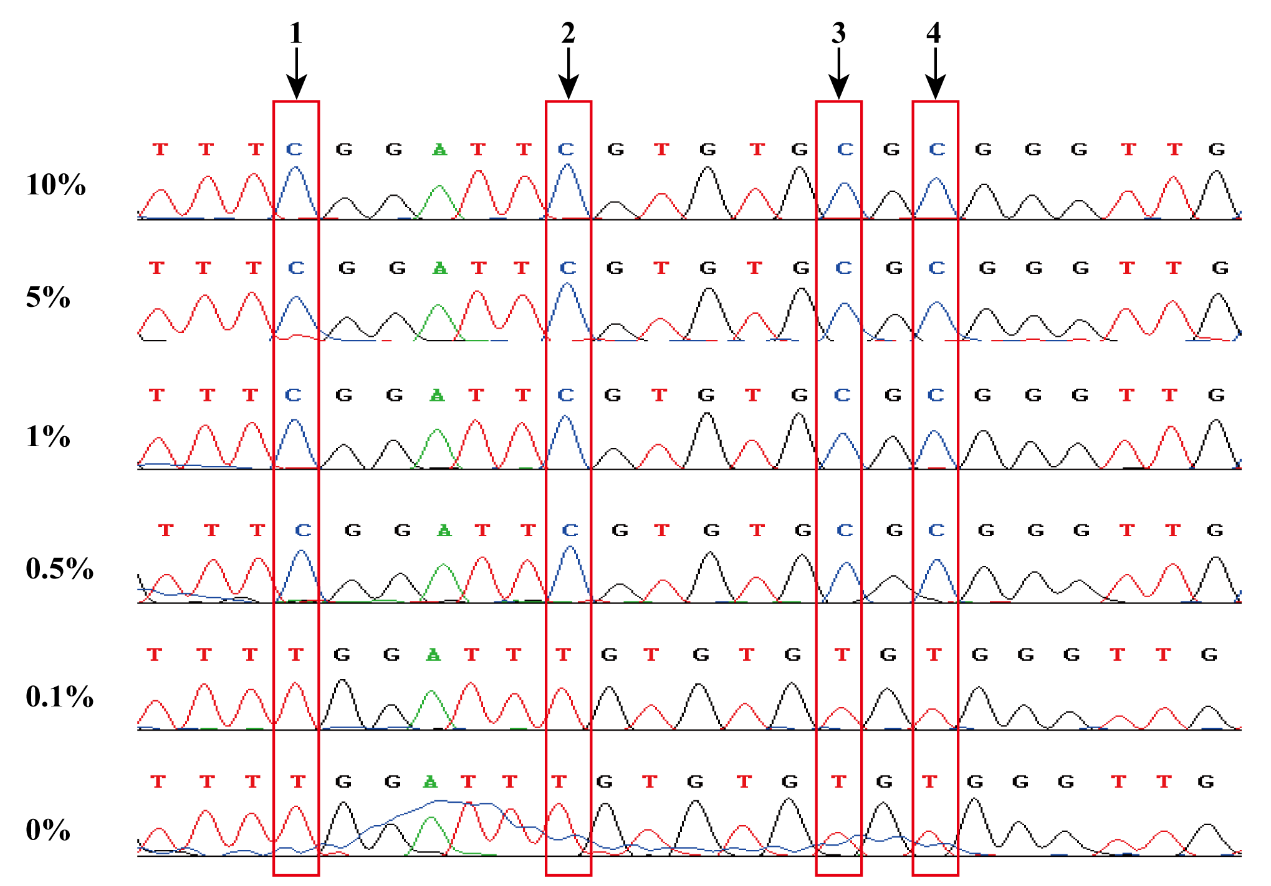


**Figure S2**. The sensitivity of BSP in detecting different methylation ratio templets. 1) the SDC2-6 site. 2-3-4) Other methylated sites.


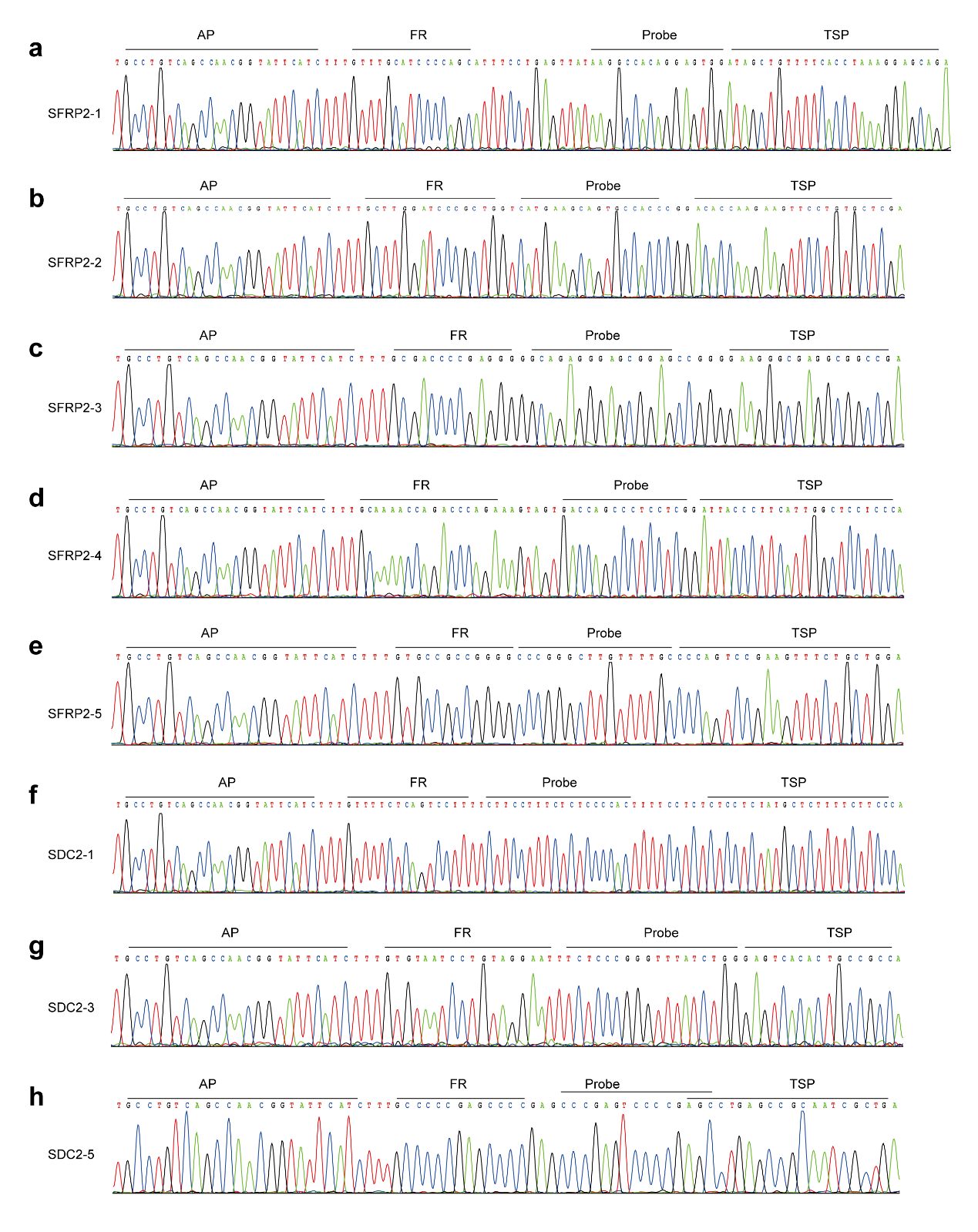


**Figure S3**. The sequence of ColoC-mSTEM PCR products detected by cloning sequencing. a-e) ColoC-mSTEM PCR products sequence of the CpG locates in *SFRP2* gene (SFRP2-1, SFRP2-2, SFRP2-3, SFRP2-4 and SFRP2-5, respectively); f-h) ColoC-mSTEM PCR products sequence of the CpG locates in *SDC2* gene (SDC2-1, SDC2-3 and SDC2-5, respectively).


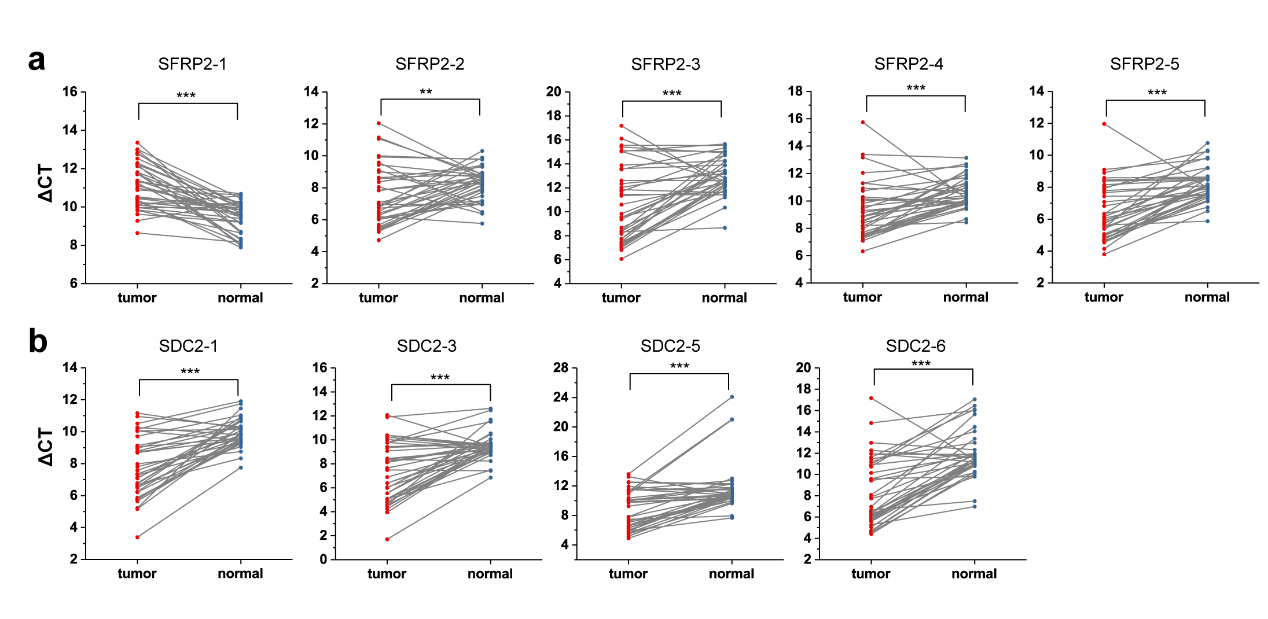


**Figure S4**. Relative methylation levels of SFRP2 and SDC2 CpG sites in 38 pairs tumor and normal tissues of patients with CRC. A) Differences in relative methylation levels of SFRP2 locates between tumor and normal tissues. B) Differences in relative methylation levels of SDC2 locates between tumor and normal tissues. ΔCT (the Ct value of target gene minus the Ct value of ACTB) represents the relative methylation level of target CpG locate, **p<0.01, *** p < 0.001.

**Table S1.** The average methylation level of CpG sites in tissues

| Name | IImnID | TCGA database | | GEO database | |
| --- | --- | --- | --- | --- | --- |
|  |  | CRC tumor (n=299) | normal (n=298) | CRC tumor (n=64) | normal  (n=64) |
| SFRP2-1 | cg10318528 | 31.4% | 78.1% | 51.3% | 70.0% |
| SFRP2-2 | cg05774801 | 50.3% | 15.5% | 55.8% | 17.4% |
| SFRP2-4 | cg03202804 | 57.7% | 9.1% | 59.4% | 17.2% |
| SFRP2-6 | cg25645268 | 50.0% | 5.1% | 41.0% | 8.1% |
| SDC2-2 | cg13096260 | 54.5% | 3.5% | 43.1% | 7.6% |
| SDC2-3 | cg24732574 | 36.8% | 4.7% | 43.0% | 7.5% |
| SDC2-4 | cg16935295 | 52.7% | 2.4% | 43.3% | 4.0% |

**Table S2.** The precision of ColoC-mSTEM assays in *SFRP2* gene

| **copies/test** | **SFRP2-1** | | **SFRP2-2** | | **SFRP2-3** | | **SFRP2-4** | | **SFRP2-5** | |
| --- | --- | --- | --- | --- | --- | --- | --- | --- | --- | --- |
|  | **avg Ct** | **CV** | **avg Ct** | **CV** | **avg Ct** | **CV** | **avg Ct** | **CV** | **avg Ct** | **CV** |
| 640 | 27.50 | 2.00% | 25.00 | 2.87% | 25.29 | 2.45% | 27.67 | 1.70% | 23.33 | 1.69% |
| 320 | 28.45 | 2.45% | 25.95 | 4.16% | 26.31 | 2.61% | 28.93 | 1.91% | 24.32 | 2.02% |
| 160 | 29.68 | 3.65% | 26.99 | 3.44% | 27.53 | 1.85% | 30.21 | 1.39% | 25.13 | 3.02% |
| 80 | 30.30 | 3.78% | 28.17 | 3.80% | 28.43 | 2.40% | 31.61 | 2.84% | 25.79 | 3.87% |
| 40 | 31.24 | 4.88% | 29.26 | 4.62% | 29.31 | 2.26% | 32.81 | 4.00% | 27.09 | 2.91% |
| blank | NA |  | NA |  | NA |  | NA |  | NA |  |

**Table S3.** The precision of ColoC-mSTEM assays in *SDC2* gene

| **copies/test** | **SDC2-1** | | **SDC2-3** | | **SDC2-5** | | **SDC2-6** | |
| --- | --- | --- | --- | --- | --- | --- | --- | --- |
|  | **avg Ct** | **CV** | **avg Ct** | **CV** | **avg Ct** | **CV** | **avg Ct** | **CV** |
| 640 | 26.59 | 1.69% | 23.14 | 2.06% | 24.94 | 1.77% | 24.74 | 0.73% |
| 320 | 28.00 | 2.25% | 24.11 | 1.52% | 27.10 | 2.81% | 25.94 | 0.97% |
| 160 | 29.47 | 2.37% | 25.14 | 1.75% | 28.60 | 3.04% | 27.06 | 1.04% |
| 80 | 30.51 | 3.54% | 26.29 | 1.59% | 30.22 | 5.10% | 28.25 | 1.82% |
| 40 | 32.31 | 4.89% | 27.19 | 1.89% | 31.00 | 5.37% | 29.23 | 2.52% |
| blank | NA |  | NA |  | NA |  | NA |  |

**Table S4.** Diagnostic Models obtained from logistic regression

| **Model** | **Model1** | **Model2** | **Model3** |
| --- | --- | --- | --- |
| Factors | SDC2-6 (Ct=a) | SDC2-6 (Ct=a) | SDC2-6 (Ct=a) |
|  | c | SFRP2-1 (Ct=b) | SFRP2-1 (Ct=b) |
|  |  | c | SDC2-1 (Ct=d) |
|  |  |  | c |
| Formula | y=-0.510*a+16.3 | y=-0.322*a+(-1.048) *b+45.631 | y=-0.174*a+(-0.224) *b+(-0.978*d) +45.831 |
| Predicate value | p=1/（1+Exp^((-1)*y)） | | |
| AUC | 0.958 | 0.972 | 0.975 |

**Table S5**. The performance of methylated DNA in stool DNA for the diagnose of CRC and AA

| Author | Method | Positive Detection Rate (%) | | specificity | ref |
| --- | --- | --- | --- | --- | --- |
|  |  | AA | CRC |  |  |
| Wang JP et al. | mSDC2 | 42.1% | 83.8% | 98% | ^[19]^ |
| Oh TJ. et al. | mSDC2 | 33% to small polyps (<10 mm) | 90% | 90.9% | ^[41]^ |
| Niu F et al. | mSDC2 | 58.2% | 81.1% | 93.3% | ^[42]^ |
| ColonClear | FIT | 36.7% | 94.6% | 97.9% | ^[8]^ |
|  | ColonClear | 53.1% | 97.5% | 89.1% |  |
| Zhang WS et al. | SDC2 | 68.8% | 77.0% | 98.1% | ^[17]^ |
|  | TFPI2 | 75% | 90.2% | 94.3% |  |
|  | SDC2&TFPI2 | 81.3% | 93.4% | 94.3% |  |
| SpecColon | mSDC2 | 46.2% | 86.2% | 97.4% | ^[18]^ |
|  | mSDC2& mSFRP2 | 61.5% | 89.7% | 89.5% |  |
|  | ColonClear | 53.1% | 97.5% | 89.1% |  |
| ColoC-mSTEM | SDC 2-6 | 58.10% | 91.2% | 95.7% | Present study |
|  | SD/SF- positive | 61.8% | 94.6% | 92.5% |  |
|  | Model 3 | 61.8% | 94.1% | 95.7% |  |

**Table S6.** The Methylation ratio of SDC2-3 and SDC2-6 detected by NGS

| ID | Control | SDC2-3 | SDC2-6 |
| --- | --- | --- | --- |
| 1 | 77.89% | 2.57% | 11.49% |
| 2 | 79.75% | 1.48% | 5.79% |
| 3 | 79.98% | 0.04% | 7.51% |
| 4 | 93.60% | 0.00% | 6.28% |
| 5 | 79.44% | 0.09% | 0.26% |
| 6 | 79.06% | 0.13% | 0.18% |
| 7 | 92.92% | 0.45% | 1.89% |
| 8 | 85.17% | 4.56% | 7.79% |
| 9 | 82.85% | 0.19% | 4.13% |
| 10 | 85.71% | 0.08% | 1.15% |
| 11 | 76.06% | 0.97% | 1.91% |
| 12 | 85.22% | 21.42% | 39.71% |
| 13 | 82.77% | 13.27% | 41.11% |
| 14 | 80.39% | 1.13% | 7.30% |
| 15 | 87.68% | 10.21% | 42.63% |
| 16 | 80.83% | 8.24% | 21.31% |
| 17 | 76.67% | 15.53% | 18.83% |
| 18 | 80.17% | 18.98% | 44.38% |
| 19 | 83.82% | 74.55% | 86.71% |
| 20 | 83.21% | 65.29% | 87.94% |
| 21 | 85.27% | 70.94% | 86.87% |
| mean |  | 14.70% | 25.00% |

**Table S7.** The information of enrolled samples

| **Characteristic** | **CRC** | **AA** | **polyp** | **enteritis** | **Normal** |
| --- | --- | --- | --- | --- | --- |
| total(n) | 205 | 31 | 18 | 14 | 161 |
| Gender |  |  |  |  |  |
| Female-no.(%) | 123(60.0%) | 18(58.1%) | 8(44.4%) | 6(42.9%) | 68(42.2%) |
| Male-no.(%) | 82(40.0%) | 13(41.9%) | 10(55.6%) | 8(57.1%) | 93(57.8%) |
| Age(years) |  |  |  |  |  |
| mean | 63 | 61 | 63 | 64 | 62 |
| Range | 35-79 | 61-73 | 51-71 | 51-76 | 51-74 |
| stage |  |  |  |  |  |
| Ⅰ | 38(18.5%) | NA | NA | NA | NA |
| Ⅱ | 77(37.6%) | NA | NA | NA | NA |
| Ⅲ | 65(31.7%) | NA | NA | NA | NA |
| Ⅳ | 14(6.8%) | NA | NA | NA | NA |
| NA | 11(5.4%) | NA | NA | NA | NA |
